# Multiscale Analysis of Bi-Layer Lattice-Filled Sandwich Structure Based on NIAH Method

**DOI:** 10.3390/ma15217710

**Published:** 2022-11-02

**Authors:** Jun Yan, Chenguang Zhang, Xin Li, Liang Xu, Zhirui Fan, Wei Sun, Guangyuan Wang, Kun Yan

**Affiliations:** 1 State Key Laboratory of Structural Analysis for Industrial Equipment, Department of Engineering Mechanics, International Research Center for Computational Mechanics, Dalian University of Technology, Dalian 116024, China; 2 Ningbo Research Institute, Dalian University of Technology, Ningbo 315016, China; 3 Shanghai Academy of Spaceflight Technology, Shanghai 201109, China; 4 College of Aerospace Engineering, Nanjing University of Aeronautics & Astronautics, Nanjing 213300, China; 5 China Academy of Space Technology, Beijing 100094, China; 6 School of Chemical Engineering, Dalian University of Technology, Dalian 116024, China

**Keywords:** Bi-layer lattices, NIAH, multiscale analysis, shear stiffness

## Abstract

Bi-layer lattice-filled sandwich structures have good application prospects for multi-physics problems; however, high-precision numerical analysis methods are lacking. Recently, the newly proposed asymptotic homogenization method called the novel numerical implementation of asymptotic homogenization (NIAH) was further developed based on the Mindlin plate theory, which is a potential method for overcoming the above limitation. This study investigates the feasibility of this method for Bi-layer lattice-filled sandwich structures. The obtained results are compared to those from homogenization methods developed based on the Kirchhoff theory, and accordingly, the influence of the shear effect on the accuracy of the structural responses of the considered structures is studied. Subsequently, the impacts of the size effect, macrostructure type, and lattice type are also considered. The analysis results showed that, for most cases, the NIAH method can yield high-precision results for Bi-layer lattice-filled sandwich structures. When the number of lattice cells is insufficient or different layers of the lattice have excessive differences in their stiffness, the accuracy of the results obtained using the NIAH method is degraded.

## 1. Introduction

Lattice-filled sandwich structures have been extensively studied and used in applications owing to their advantages, such as ultrahigh specific stiffness and specific strength [1,2,3,4,5]. However, conventional lattice structures typically consist of uniform and consistent lattices, which limits the performance of lattice-filled structures. Recently, inspired by natural structures, some studies proposed the concept of a gradient lattice [6] and found that gradient lattice structures show improved performance in structural multi-physics fields compared to traditional types [7,8]. 

In a gradient lattice-filled sandwich structures, the lattice shows regular changes in the thickness direction, which may be variations in the lattice structure or the lattice size, whereas the lattice remains unchanged in the plane direction. 

Research on gradient lattices has been conducted in many fields [9,10,11]. Zhang et al. [12] performed a simulation analysis of the impact properties of honeycomb materials, proposing that the introduction of a density gradient affects their overall performance. Lefebvre et al. [13] used an analytical method to analyse the wave propagation problem of functionally graded plates. Sun et al. [14] studied the dynamic response of uniform and graded foamy aluminium core materials under the influence of an impact load by conducting tests and simulations. They found that the gradient structure had a significant effect on both the structural deformation and failure of these materials. Zhou et al. [15] performed impact tests on sandwich structures and demonstrated the superiority of gradient structures. Woodward et al. [16] conducted a three-dimensional elastic analysis of sandwich plates and comparatively studied the effect of stress on gradient core plates and uniform sandwich plates under different load distributions. Liu et al. [17] investigated the two-power response and implosion performance of sandwich and non-gradient laminated plates containing functionally graded foamy aluminium core materials by conducting tests and finite element (FE) simulations. They concluded that the structural performance can be further improved by optimizing the core layer arrangement. Xu et al. [18] proposed a lattice sandwich structure with a gradient in the in-plane direction, solved it analytically, and tested its mechanical properties under bending loads. Based on a segmented exponential model developed by Guo et al. [19] and related research, an analytical method was proposed for the correlation analysis of gradient lattice structures, and it was found to be feasible for analysing thermal problems. Ajdari et al. [20] showed that the introduction of a density gradient significantly changes the deformation pattern and the energy absorption of a honeycomb structure at both low and high breaking speeds. Cheng et al. [21] designed a three-dimensional gradient lattice structure by topological optimization under stress constraints, showing that the mechanical properties of the optimized structure were remarkably improved compared to those of the homogeneous lattice. Although Li et al. [22] conducted a multiscale optimization design of gradient porous materials based on a homogenization method, it was difficult to meet the periodic boundary condition using such a technique in the structure height direction. The above work is concerned with load bearing, impact resistance and other properties, and fatigue-related applications are also worth researching. The gradient lattice structures are often used for shell, so it is necessary to pay attention to the global deformation in structural analysis. One problem hindering the application of gradient lattice structures is the lack of efficient and high-precision analysis methods leading to their structural response analysis, typically requiring conducting direct analysis using finite element models. Because gradient lattice structures contain many details, their finite element models are typically very large, making their direct analysis highly time consuming. However, there are few papers on multi-scale analysis of such structures. 

In uniform lattice-filled sandwich structures, this problem has been overcome using homogenization analysis methods. Homogenization methods treat a uniform lattice-filled sandwich structure as a solid structure with material parameters that depend on the used lattice structure. However, most conventional homogenization methods use the Kirchhoff theory, and thus, lack the consideration of the shear effects. Concurrently, significant shear deformation occurs in the structural response of a gradient lattice structure. For a gradient lattice sandwich plate, the lattice structure changes in the thickness direction. Thus, the gradient lattice plate structure no longer strictly satisfies the assumption of a straight line. If the Kirchhoff plate assumption is still adopted, the structural responses would be smaller than the true responses because of the overestimation of the shear stiffness. Thus, the Mindlin plate theory is expected to be relatively more suitable for gradient lattice-filled sandwich structures.

With the recent developments in the novel implementation of asymptotic homogenization (NIAH) method, shear stiffness can be introduced in homogenization analysis, providing a potentially effective method for solving the above-mentioned problem. The NIAH method was first proposed by Cheng [23] and is based on the theory of perturbation expansion. With using a novel numerical implementation of asymptotic homogenization (NIAH) method, Wang et al. [24] proposed an effective and efficient numerical-based smeared stiffener method (NSSM) for the buckling analysis of grid-stiffened composite cylindrical shells. For plate structures, in which it is difficult to meet the requirements of the periodicity of the lattice structure in the thickness direction, the solution format with periodic asymptotically homogenization in three dimensions produces large errors [25]. Based on the NIAH method, Cai et al. [26] developed a progressive homogenization numerical solution for the equivalent properties of two-dimensional plate problems based on the Kirchhoff theory. On its basis, Xu et al. [27] introduced a shear term into the NIAH method by adopting the Mindlin assumption, thereby establishing a general and effective method for predicting the equivalent shear stiffness, which can be implemented using the FE method (FEM).

**Figure 1:**
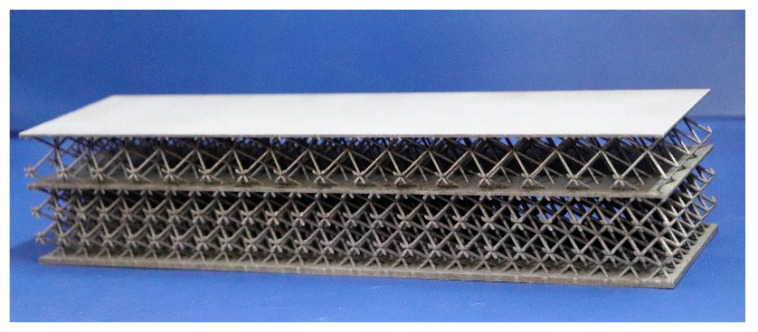
Bi-layer lattice-filled sandwich structure by additive manufacturing [28].

Compared to gradient lattice structures, Bi-layer lattice-filled sandwich structures, as shown as Figure 1, retain the variation of gradient in *z* direction but also dictate the size of each layer of the lattice uniform due to Bi-layer so that it can easily be analysed and manufactured. Therefore, it is often used in practical engineering applications. This study takes the Bi-layer lattice-filled sandwich structure as an example to investigate the feasibility of the NIAH method for the structural analysis of gradient lattice structures, which solves the problem of the lack of efficient and high-accuracy analysis methods for these structures. The effectiveness of the proposed method to solve the structural equivalence analysis of a Bi-layer lattice-filled sandwich structure is verified based on various examples. 

The remainder of this paper is as follows. Section 2 introduces the equivalent stiffness prediction method of a periodic plate structure based on the NIAH. The effects of unit cell parameters and different unit cells on the equivalent analysis are discussed by numerical examples in Section 3 and Section 4, respectively. The conclusion of this study is given in Section 5.

## 2. Analysis of Structural Equivalent Stiffness

### 2.1. Calculation of Equivalent Stiffness Using NIAH Method

**Figure 2:**
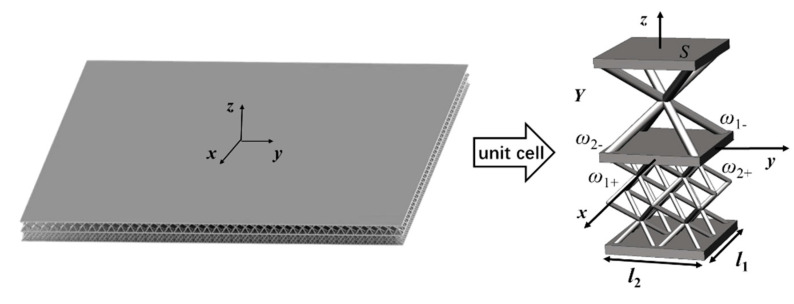
Bi-layer lattice-filled sandwich plate and its unit cell.

For a Bi-layer lattice-filled sandwich plate structure, as shown in Figure 2, a unit cell is defined as $Y=\left\{\left(x,y,z\right)|-l_{1}/2\leq x\leq l_{1}/2,-l_{2}/2\leq y\leq l_{2}/2\right\}$. The periodic boundary is $\omega_{1\pm }$ in the *x* direction and $\omega_{2\pm }$ in the *y* direction, and the aperiodic boundary is *S*.

Using the NIAH method, the equivalent stiffness of one unit cell of the Bi-layer lattice-filled sandwich plate can be obtained in three steps.

In the first step, the displacement field, which generates the unit generalized strain field, is calculated. For the periodic plate structure, the unit generalized strains are defined as

(1) $$\varepsilon =\left\{\begin{array}{c} \varepsilon_{11}\\ \varepsilon_{22}\\ \varepsilon_{33}\\ \gamma_{12}\\ \gamma_{23}\\ \gamma_{31} \end{array}\right\},\;\;\varepsilon^{1}=\left\{\begin{array}{c} 1\\ 0\\ 0\\ 0\\ 0\\ 0 \end{array}\right\},\;\;\varepsilon^{2}=\left\{\begin{array}{c} 0\\ 1\\ 0\\ 0\\ 0\\ 0 \end{array}\right\},\;\;\varepsilon^{3}=\left\{\begin{array}{c} 0\\ 0\\ 0\\ 1\\ 0\\ 0 \end{array}\right\},\varepsilon^{4}=\left\{\begin{array}{c} z\\ 0\\ 0\\ 0\\ 0\\ 0 \end{array}\right\},\;\;\varepsilon^{5}=\left\{\begin{array}{c} 0\\ z\\ 0\\ 0\\ 0\\ 0 \end{array}\right\},\;\;\varepsilon^{6}=\left\{\begin{array}{c} 0\\ 0\\ 0\\ z\\ 0\\ 0 \end{array}\right\}$$

where $\varepsilon^{1}$ and $\varepsilon^{2}$ denote the unit membrane strains along the *x* and *y* directions, respectively, $\varepsilon^{3}$ denotes the in-plane unit shear strain, $\varepsilon^{4}$ and $\varepsilon^{5}$ denote the unit bending strains, respectively, and $\varepsilon^{6}$ denotes the unit torsional strain. 

The corresponding characteristic displacement fields in the unit cell equivalent to the six generalized unit strains can be obtained using

(2) $$\begin{array}{l} \chi =\left\{\begin{array}{l} \chi_{1}\\ \chi_{2}\\ \chi_{3}\\ \theta_{1}\\ \theta_{2}\\ \theta_{3} \end{array}\right\},\;\;\chi^{1}=\left\{\begin{array}{c} x\\ 0\\ 0\\ 0\\ 0\\ 0 \end{array}\right\},\;\;\chi^{2}=\left\{\begin{array}{c} 0\\ y\\ 0\\ 0\\ 0\\ 0 \end{array}\right\},\;\;\chi^{3}=\left\{\begin{array}{c} y/2\\ x/2\\ 0\\ 0\\ 0\\ 0 \end{array}\right\},\;\;\\ \chi^{4}=\left\{\begin{array}{c} zx\\ 0\\ -x^{2}/2\\ 0\\ x\\ 0 \end{array}\right\},\;\;\chi^{5}=\left\{\begin{array}{c} 0\\ zy\\ -y^{2}/2\\ -y\\ 0\\ 0 \end{array}\right\},\;\;\chi^{6}=\left\{\begin{array}{c} zy/2\\ zx/2\\ -xy/2\\ -x/2\\ y/2\\ 0 \end{array}\right\} \end{array}$$

where $\chi_{i}$ (*i* = 1, 2, 3) represents the node displacement along the *x*, *y* or *z* direction and $\theta_{i}$ (*i* = 1, 2, 3) represents the nodes angle of rotation along the *x*, *y* or *z* direction. 

In the second step, six independent structural analyses must first be performed on a unit lattice cell using the six displacements obtained in the first step, respectively. The node forces $f^{\alpha }$ (*α* = 1, 2, …, 6) from the structural analysis are the results that are to be obtained from these structural analyses. The governing equations of these structural analyses are expressed in Equation (3).

(3) $$\left\{ \begin{array}{l} \frac{\partial }{\partial y_{i}}\left(c_{ijkl}\left(\frac{\partial {\tilde{\chi }}_{k}^{\alpha }}{\partial y_{l}}+\varepsilon_{kl}^{\alpha }\right)\right)=0\;\;\;in\;Y\\ {c_{ijkl}\left(\frac{\partial {\tilde{\chi }}_{k}^{\alpha }}{\partial y_{l}}+\varepsilon_{kl}^{\alpha }\right)n_{j}|}_{S}=0\;\;\;on\;S\\ {\left(c_{ijkl}\frac{\partial {\tilde{\chi }}_{k}^{\alpha }}{\partial y_{l}}\right)n_{j}|}_{\omega_{1+}}=-{\left(c_{ijkl}\frac{\partial {\tilde{\chi }}_{k}^{\alpha }}{\partial y_{l}}\right)n_{j}|}_{\omega_{1-}}\;\;\;on\;\omega_{1\pm }\\ {\left(c_{ijkl}\frac{\partial {\tilde{\chi }}_{k}^{\alpha }}{\partial y_{l}}\right)n_{j}|}_{\omega_{2+}}=-{\left(c_{ijkl}\frac{\partial {\tilde{\chi }}_{k}^{\alpha }}{\partial y_{l}}\right)n_{j}|}_{\omega_{2-}}\;\;\;on\;\omega_{2\pm }\\ {{\tilde{\chi }}_{k}^{\alpha }|}_{\omega_{1+}}={{\tilde{\chi }}_{k}^{\alpha }|}_{\omega_{1-}}\;\;\;on\;\omega_{1\pm }\\ {{\tilde{\chi }}_{k}^{\alpha }|}_{\omega_{2+}}={{\tilde{\chi }}_{k}^{\alpha }|}_{\omega_{2-}}\;\;\;on\;\omega_{2\pm } \end{array}\right.$$

The first equation of Equation (3) denotes body force in the unit cell domain ***Y*** as constant. The second denotes tractions on nonperiodic boundaries ***S*** as zero. Others are the periodic boundary conditions of force and displacement on the two-direction boundary in the plane. Subsequently, the node force $-f^{\alpha }$ is used to analyse the FEM model of the unit cell, and the displacement field ${\tilde{\chi }}^{\alpha }$ (*α* = 1, 2, …, 6) is computed by applying the boundary conditions of the displacement and the force and restricting the rigid body displacement. Subsequently, the displacement field is reloaded on the unit cell to obtain the corresponding node forces, ${\tilde{f}}^{\alpha }$ (*α* = 1, 2, …, 6). 

**Figure 3:**
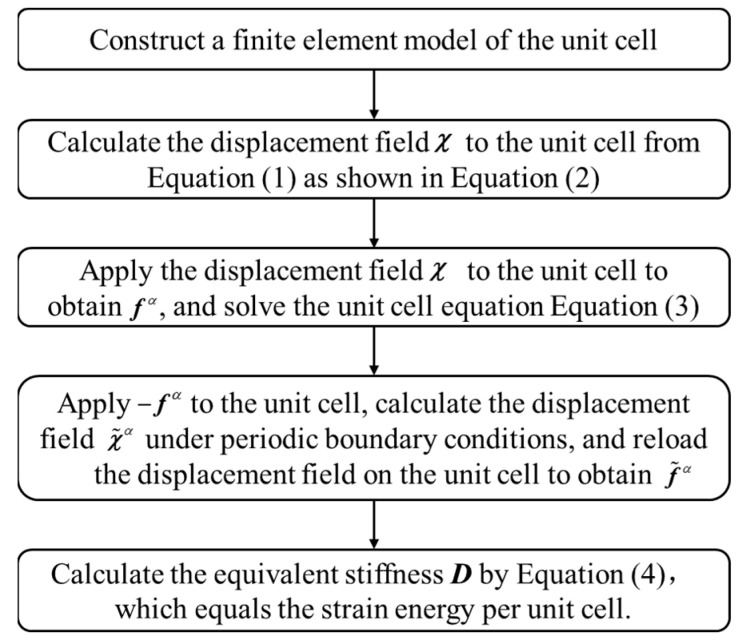
Flowchart of NIAH method to calculate equivalent stiffness.

Finally, in the third step, the equivalent stiffness, ***D***, is determined by solving Equation (4). A flowchart of the NIAH method for solving equivalent stiffness ***D*** is shown in Figure 3.

(4) $$\begin{array}{l} D=\left[\begin{array}{cccccc} D_{11} & D_{12} & D_{13} & D_{14} & D_{15} & D_{16}\\ D_{21} & D_{22} & D_{23} & D_{24} & D_{25} & D_{26}\\ D_{31} & D_{32} & D_{33} & D_{34} & D_{35} & D_{36}\\ D_{41} & D_{42} & D_{43} & D_{44} & D_{45} & D_{46}\\ D_{51} & D_{52} & D_{53} & D_{54} & D_{55} & D_{56}\\ D_{61} & D_{62} & D_{63} & D_{64} & D_{65} & D_{66} \end{array}\right]\\ D_{\alpha \beta }=\frac{1}{\left|Y\right|}{\left(\chi^{\alpha }+{\tilde{\chi }}^{\alpha }\right)}^{T}\left(f^{\beta }+{\tilde{f}}^{\beta }\right){}^{}{}^{}\left(\alpha ,\beta =1,\;2,\;\ldots ,\;6\right) \end{array}$$

### 2.2. Calculation of Shear Stiffness Using NIAH Method

**Figure 4:**
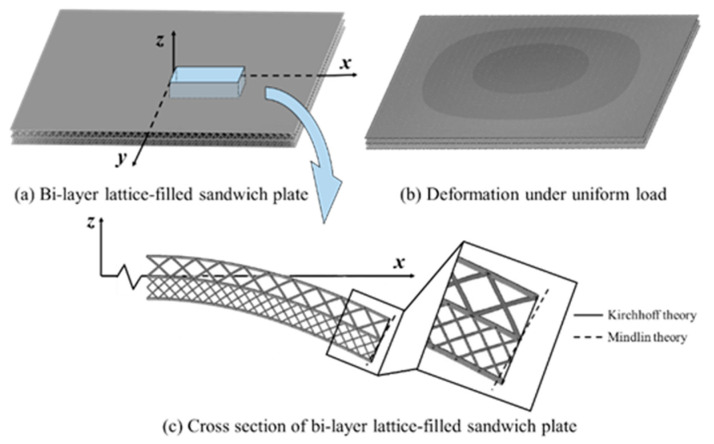
Deformation mode of Bi-layer lattice-filled sandwich plate under uniform load.

For a Bi-layer lattice-filled sandwich plate, the mode of bending deformation does not typically strictly follow the assumptions of a straight line, as shown in Figure 4. Consequently, the predicted structural shear stiffness based on the Kirchhoff theory may be larger than the actual one, i.e., the structural deformation may be underestimated. Specifically, for a Bi-layer lattice-filled plate, the Mindlin plate theory needs to be adopted to obtain high-precision structural responses.

The calculation steps of the NIAH method based on the Mindlin plate theory (S-NIAH) includes those of the NIAH method, as shown in Figure 3, and additional steps to obtain the shear stiffness. 

It is assumed that a Bi-layer lattice-filled plate satisfies the matrix form of the constitutive equation

(5) $$\begin{array}{l} \left\{\begin{array}{l} N\\ M \end{array}\right\}=\left[\begin{array}{cc} D^{1} & D^{2}\\ D^{2}{}^{T} & D^{4} \end{array}\right]\left\{\begin{array}{l} \varepsilon \\ \kappa \end{array}\right\},\;\;Q=K\gamma \\ D^{1}=\left[\begin{array}{ccc} D_{11} & D_{12} & D_{13}\\ D_{21} & D_{22} & D_{23}\\ D_{31} & D_{32} & D_{33} \end{array}\right]{}^{}D^{2}=\left[\begin{array}{ccc} D_{14} & D_{15} & D_{16}\\ D_{24} & D_{25} & D_{26}\\ D_{34} & D_{35} & D_{36} \end{array}\right]{}^{}D^{4}=\left[\begin{array}{ccc} D_{44} & D_{45} & D_{46}\\ D_{54} & D_{55} & D_{56}\\ D_{64} & D_{65} & D_{66} \end{array}\right] \end{array}$$

where ***D***^1^, ***D***^2^, and ***D***^4^ represent the in-plane stiffness, coupling stiffness, and bending stiffness, respectively; ***N*** and ***M*** are the in-plane internal force and the bending moment corresponding to strain ε and curvature κ; ***Q*** is the shear force and corresponds to the shear deformations of the plate γ; and ***K*** is the equivalent shear stiffness. Note that in Equation (5), only the shear stiffness matrix, ***K***, still has no calculation formula, and the other terms can be obtained using the equations presented in Section 2.1. 

In the S-NIAH method, the matrix, ***K***, is solved by macroscopic and microscopic strain energy equivalence. To construct the macroscopic stress–strain state related to the equivalent shear stiffness, it is assumed that in-plane internal force is ***N*** = 0 first; thus,

(6) $$\begin{array}{l} \varepsilon =-{\left(D^{1}\right)}^{-1}D^{2}\kappa \\ M=\left(D^{4}-D^{2}{}^{T}{\left(D^{1}\right)}^{-1}D^{2}\right)\kappa \end{array}$$

Equation (6) can be rewritten as

(7) $$\begin{array}{l} \varepsilon =F\kappa \\ M=\bar{D}\kappa \end{array}$$

where $F=-{\left(D^{1}\right)}^{-1}D^{2},\bar{D}=D^{4}-D^{2}{}^{T}{\left(D^{1}\right)}^{-1}D^{2}$. A plate element of macroscopic size *L*_1_ × *L*_2_ is chosen, and a strain field of linear curvature is defined as $\kappa_{11}$ (Equation (8)) for the chosen plate element.

(8) $$\left\{\begin{array}{c} \kappa_{11}\\ \kappa_{22}\\ \kappa_{12} \end{array}\right\}=\left\{\begin{array}{c} 1\\ 0\\ 0 \end{array}\right\}\frac{x_{1}}{L_{1}},\;\;\left\{\begin{array}{c} \varepsilon_{11}\\ \varepsilon_{22}\\ \gamma_{12} \end{array}\right\}=\left\{\begin{array}{c} F_{11}\\ F_{21}\\ F_{31} \end{array}\right\}\frac{x_{1}}{L_{1}},\;\;\left\{\begin{array}{l} \gamma_{13}\\ \gamma_{23} \end{array}\right\}=\left\{\begin{array}{l} \frac{{\bar{D}}_{11}}{L_{1}K_{11}}\\ \frac{{\bar{D}}_{13}}{L_{1}K_{22}} \end{array}\right\}$$

Equation (8) can be brought back to Equation (6) and the equilibrium equation to obtain Equation (9).

(9) $$N=\left\{\begin{array}{c} 0\\ 0\\ 0 \end{array}\right\},\;\;M=\left\{\begin{array}{c} {\bar{D}}_{11}\\ {\bar{D}}_{12}\\ {\bar{D}}_{13} \end{array}\right\}\frac{x_{1}}{L_{1}},\;\;Q=\left\{\begin{array}{l} \frac{{\bar{D}}_{11}}{L_{1}}\\ \frac{{\bar{D}}_{13}}{L_{1}} \end{array}\right\}$$

The macroscopic strain energy at this strain state by $e=\frac{1}{2}\int \left(N\varepsilon +M\kappa +Q\gamma \right)dA$ is

(10) $$e=\frac{{\bar{D}}_{11}L_{1}L_{2}}{24}+\frac{L_{2}}{2L_{1}}\left(\frac{{\bar{D}}_{11}^{2}}{K_{11}}+\frac{{\bar{D}}_{13}^{2}}{K_{22}}\right)$$

Thus, at the microscopic level, a linear displacement field $\chi_{\;}^{s1}$ corresponding to the pure shear state with linear curvature $\kappa_{11}$ is constructed, which is expressed in Equation (11).

(11) $$\begin{array}{c} \chi^{s1}=F_{11}\chi^{l1}+F_{21}\chi^{l2}+F_{31}\chi^{l3}+\chi^{l4}\\ \chi^{l1}=\left\{\begin{array}{c} y_{1}^{2}/2l\\ 0\\ 0\\ 0\\ 0\\ 0 \end{array}\right\},\;\;\chi^{l2}=\left\{\begin{array}{c} -y_{2}^{2}/2l_{1}\\ y_{1}y_{2}/l_{1}\\ 0\\ 0\\ 0\\ 0 \end{array}\right\},\;\;\chi^{l3}=\left\{\begin{array}{c} 0\\ y_{1}^{2}/2l_{1}\\ 0\\ 0\\ 0\\ 0 \end{array}\right\},\;\;\chi^{l4}=\left\{\begin{array}{c} y_{3}y_{1}^{2}/2l_{1}\\ 0\\ -y_{1}^{3}/6l_{1}\\ 0\\ y_{1}^{2}/2l_{1}\\ 0 \end{array}\right\} \end{array}$$

$\chi^{l1},\chi^{l2},\chi^{l3},\chi^{l4}$ in Equation (11) is the displacement field equivalent to the linear strain $\varepsilon_{11},\varepsilon_{22},\gamma_{12},\kappa_{11}$ along the *x* direction. To satisfy the condition that the external force equals to zero and the continuity of the displacement and the force in the periodic boundary condition, the displacement field, ${\tilde{\chi }}_{\;}^{s1}$, needs to be superimposed; therefore, the state equation is

(12) $$\left\{ \begin{array}{l} \frac{\partial }{\partial y_{j}}\left(c_{ijkl}\frac{\partial \left(\chi_{k}^{s1}+{\tilde{\chi }}_{k}^{s1}\right)}{\partial y_{l}}\right)=0\;\;\;in\;Y\\ {c_{ijkl}\frac{\partial \left(\chi_{k}^{s1}+{\tilde{\chi }}_{k}^{s1}\right)}{\partial y_{l}}n_{j}|}_{S}=0\;\;\;on\;S\\ {\left(c_{ijkl}\frac{\partial {\tilde{\chi }}_{k}^{s1}}{\partial y_{l}}\right)n_{j}|}_{\omega_{1+}}+{\left(c_{ijkl}\frac{\partial {\tilde{\chi }}_{k}^{s1}}{\partial y_{l}}\right)n_{j}|}_{\omega_{1-}}=\;{\left(c_{ijkl}\frac{\partial {\tilde{\chi }}_{k}^{b1}}{\partial y_{l}}\right)n_{j}|}_{\omega_{1+}}\;\;on\;\omega_{1\pm }\\ {{\tilde{\chi }}_{i}^{s1}|}_{\omega_{1+}}-{{\tilde{\chi }}_{i}^{s1}|}_{\omega_{1-}}\;={{\tilde{\chi }}_{i}^{b1}|}_{\omega_{1+}}\;\;on\;\omega_{1\pm }\\ {\left(c_{ijkl}\frac{\partial {\tilde{\chi }}_{k}^{s1}}{\partial y_{l}}\right)n_{j}|}_{\omega_{2+}}+{\left(c_{ijkl}\frac{\partial {\tilde{\chi }}_{k}^{s1}}{\partial y_{l}}\right)n_{j}|}_{\omega_{2-}}=\;0\;\;on\;\omega_{2\pm }\\ {{\tilde{\chi }}_{i}^{s1}|}_{\omega_{2+}}-{{\tilde{\chi }}_{i}^{s1}|}_{\omega_{2-}}\;=0\;\;on\;\omega_{2\pm } \end{array}\right.$$

where ${\tilde{\chi }}_{\;}^{b1}=F_{11}{\tilde{\chi }}_{\;}^{1}+F_{21}{\tilde{\chi }}_{\;}^{2}+F_{31}{\tilde{\chi }}_{\;}^{3}+{\tilde{\chi }}_{\;}^{4}$ is the displacement field. Under the continuity condition of force and displacement, the boundary satisfies the following relation.

(13) f˜ω1+is1+f˜ω1−is1=−f˜ω1−ib1=f˜ω1+ib1

(14) $${{\tilde{\chi }}_{\;}^{s1}|}_{\omega_{1+}}-{{\tilde{\chi }}_{\;}^{s1}|}_{\omega_{1-}}={{\tilde{\chi }}_{\;}^{b1}|}_{\omega_{1-}}={{\tilde{\chi }}_{\;}^{b1}|}_{\omega_{1+}}$$

Defining ${\bar{\chi }}^{s1}=\chi^{s1}+{\tilde{\chi }}^{s1}$ and introducing ${\tilde{\chi }}^{s1}={\bar{\chi }}^{s1}-\chi^{s1}$ into Equation (12), the governing equation (Equation (15)) for the calculation of ${\bar{\chi }}^{s1}$ is obtained.

(15) $$\left\{ \begin{array}{l} \frac{\partial }{\partial y_{j}}\left(c_{ijkl}\frac{\partial {\bar{\chi }}_{k}^{s1}}{\partial y_{l}}\right)=0\;\;\;in\;Y\\ {c_{ijkl}\frac{\partial {\bar{\chi }}_{k}^{s1}}{\partial y_{l}}n_{j}|}_{S}=0\;\;\;on\;S\\ {\left(c_{ijkl}\frac{\partial {\bar{\chi }}_{k}^{s1}}{\partial y_{l}}\right)n_{j}|}_{\omega_{1+}}+{\left(c_{ijkl}\frac{\partial {\bar{\chi }}_{k}^{s1}}{\partial y_{l}}\right)n_{j}|}_{\omega_{1-}}=\;{\left(c_{ijkl}\frac{\partial {\tilde{\chi }}_{k}^{b1}}{\partial y_{l}}\right)n_{j}|}_{\omega_{1+}}\;\;on\;\omega_{1\pm }\\ {{\bar{\chi }}_{i}^{s1}|}_{\omega_{1+}}-{{\bar{\chi }}_{i}^{s1}|}_{\omega_{1-}}\;={{\tilde{\chi }}_{i}^{b1}|}_{\omega_{1+}}+{\Delta \chi_{i}^{s1}|}_{\omega_{1}}\;\;on\;\omega_{1\pm }\\ {\left(c_{ijkl}\frac{\partial {\bar{\chi }}_{k}^{s1}}{\partial y_{l}}\right)n_{j}|}_{\omega_{2+}}+{\left(c_{ijkl}\frac{\partial {\bar{\chi }}_{k}^{s1}}{\partial y_{l}}\right)n_{j}|}_{\omega_{2-}}=\;0\;\;on\;\omega_{2\pm }\\ {{\bar{\chi }}_{i}^{s1}|}_{\omega_{2+}}-{{\bar{\chi }}_{i}^{s1}|}_{\omega_{2-}}\;={\Delta \chi_{i}^{s1}|}_{\omega_{2}}\;\;on\;\omega_{2\pm } \end{array}\right.$$

where

(16) $$\begin{array}{l} {\Delta \chi_{\;}^{s1}|}_{\omega_{1}}={\chi_{\;}^{s1}|}_{\omega_{1+}}-{\chi_{\;}^{s1}|}_{\omega_{1-}}=\left\{\begin{array}{c} 0\\ F_{21}y_{2}\\ 0\\ 0\\ 0\\ 0 \end{array}\right\}+\left\{\begin{array}{c} 0\\ 0\\ l_{1}^{2}/24\\ 0\\ 0\\ 0 \end{array}\right\}\\ {\Delta \chi_{\;}^{s1}|}_{\omega_{2}}={\chi_{\;}^{s1}|}_{\omega_{2+}}-{\chi_{\;}^{s1}|}_{\omega_{2-}}=\left\{\begin{array}{c} 0\\ \frac{F_{21}l_{2}}{l_{1}}y_{1}\\ 0\\ 0\\ 0\\ 0 \end{array}\right\} \end{array}$$

The solution of the displacement field, ${\bar{\chi }}^{s1}$, is related to the method constraining the rigid body displacement when solving ${\tilde{\chi }}^{b1}$. Assuming that a ${\tilde{\chi }}^{*b1}$ exists that creates the displacement field, ${\bar{\chi }}^{*s1}$ corresponds to a macroscopically pure shear state, and ${\bar{\chi }}^{*s1}$ can be expressed as

(17) $${\bar{\chi }}_{\;}^{*s1}={\bar{\chi }}_{\;}^{s1}+b_{1}{\bar{\chi }}_{\;}^{1}+b_{2}{\bar{\chi }}_{\;}^{3}$$

where coefficients *b*_1_ and *b*_2_ are determined using Equation (18).

(18) $$\left[\begin{array}{cc} D_{11}^{1} & D_{13}^{1}\\ D_{13}^{1} & D_{33}^{1} \end{array}\right]\left\{\begin{array}{l} b_{1}\\ b_{2} \end{array}\right\}=\left\{\begin{array}{l} -\frac{1}{l_{1}l_{2}}\int_{Y}c_{ijkl}{\bar{\varepsilon }}_{ij}^{s1}{\bar{\varepsilon }}_{ij}^{1}d\Omega \\ -\frac{1}{l_{1}l_{2}}\int_{Y}c_{ijkl}{\bar{\varepsilon }}_{ij}^{s1}{\bar{\varepsilon }}_{ij}^{3}d\Omega \end{array}\right\}$$

where ${\bar{\varepsilon }}_{ij}^{*s1}=0.5\left({\bar{\chi }}_{i,j}^{*s1}+{\bar{\chi }}_{j,i}^{*s1}\right)$. After obtaining the displacement field, ${\bar{\chi }}^{*s1}$, the following equations can be formulated:

(19) $$\frac{{\bar{D}}_{11}l_{1}l_{2}}{24}+\frac{l_{2}}{2l_{1}}\left(\frac{{\bar{D}}_{11}^{2}}{K_{11}}+\frac{{\bar{D}}_{13}^{2}}{K_{22}}\right)=\frac{1}{2}\int_{Y}c_{ijkl}{\bar{\varepsilon }}_{ij}^{*s1}{\bar{\varepsilon }}_{kl}^{*s1}d\Omega$$

Similar to the above steps (Equations (8)–(19)), Equation (20) is obtained for the strain state of linear curvature $\kappa_{22}$.

(20) $$\frac{{\bar{D}}_{22}l_{1}l_{2}}{24}+\frac{l_{1}}{2l_{2}}\left(\frac{{\bar{D}}_{23}^{2}}{K_{11}}+\frac{{\bar{D}}_{22}^{2}}{K_{22}}\right)=\frac{1}{2}\int_{Y}c_{ijkl}{\bar{\varepsilon }}_{ij}^{*s2}{\bar{\varepsilon }}_{kl}^{*s2}d\Omega$$

**Figure 5:**
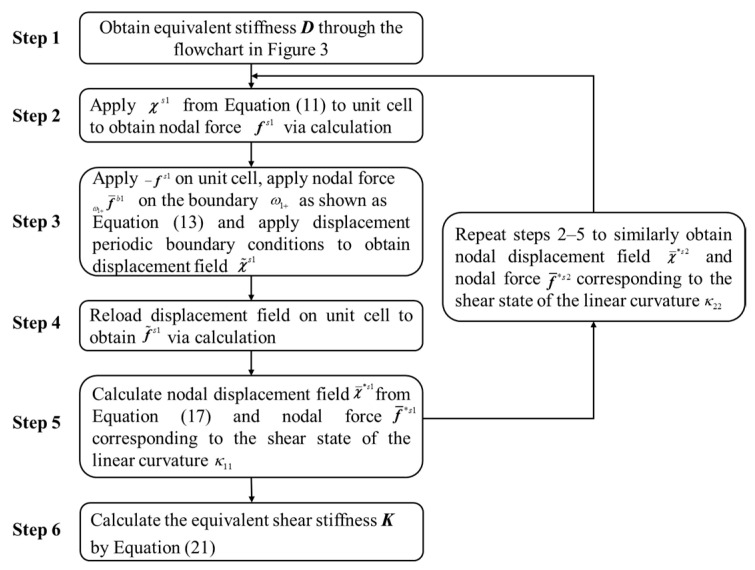
Flowchart of S-NIAH method to solve equivalent shear stiffness.

Combining Equations (19) and (20), the equivalent shear stiffness coefficients *K*_11_ and *K*_22_ can be solved. The FE forms of Equations (19) and (20) are expressed in Equation (21), and the process of derivation is seen in the literature [27]. A flowchart of the S-NIAH method for solving equivalent shear stiffness ***K*** is shown in Figure 5.

(21) $$\begin{array}{c} \frac{{\bar{D}}_{11}l_{1}l_{2}}{24}+\frac{l_{2}}{2l_{1}}\left(\frac{{\bar{D}}_{11}^{2}}{K_{11}}+\frac{{\bar{D}}_{13}^{2}}{K_{22}}\right)=\frac{1}{2}{\left({\bar{\chi }}^{*s1}\right)}^{T}{\bar{f}}^{*s1}\\ \frac{{\bar{D}}_{22}l_{1}l_{2}}{24}+\frac{l_{1}}{2l_{2}}\left(\frac{{\bar{D}}_{23}^{2}}{K_{11}}+\frac{{\bar{D}}_{22}^{2}}{K_{22}}\right)=\frac{1}{2}{\left({\bar{\chi }}^{*s2}\right)}^{T}{\bar{f}}^{*s2} \end{array}$$

## 3. Numerical Examples of the Effect of Unit Cell Parameters on Equivalent Analysis

In this study, we performed equivalent analysis of a Bi-layer lattice-filled structure using ANSYS, in which the lattice unit cell, as shown in Figure 2, is modelled using a beam element and shell element. The equivalent stiffness, ***D***, and the equivalent shear stiffness, ***K***, can be obtained based on the flowcharts shown in Figure 3 and Figure 5. Following this, the equivalent model is modelled using shell elements in the equivalent analysis and the material properties are obtained.

### 3.1. Calculation of Equivalent Stiffness of Single Bi-layer Lattice Cell

**Figure 6:**
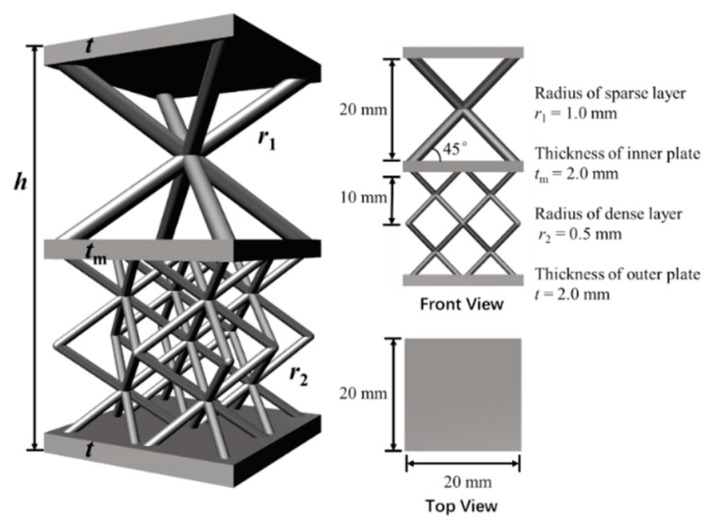
Schematic of Bi-layer lattice sandwich unit cell.

Body centred cubic (BCC) lattices as a positive Poisson’s ratio structure show high strength over a wide range of temperatures and large strain states. The considered example is a single Bi-layer lattice unit cell, as shown in Figure 6. The Bi-layer lattice structure is composed of two lattices layers: sparse and dense layers. However, the sizes of the infilled lattices are different. The height of each layer is 20 mm. The radii of all robs in the sparse layer, *r*_1_, are set as 1.0 mm each and in the dense layer, *r*_2_, are set as 0.5 mm each. The thickness, *t*, of the plates on the upper and lower bounds is 2.0 mm, and the thickness, *t*_m_, of the inner plates is also 2.0 mm. The material parameters of aluminium are used with Young’s modulus is 71,000 MPa and Poisson’s ratio is 0.33.

Using the NIAH method, the equivalent stiffness, ***D***, of the unit cell can be calculated following the steps given in Figure 2, and the result is expressed in Equation (22). Bi-layer lattice-filled sandwich plate is equated as an orthotropic plate. Since the midplane coordinate *z* = 0, there is no coupling stiffness and each element of ***D***^2^ is zero, and there is no tensile-shear coupling or bending-torsional coupling, so $D_{16}^{1}$, $D_{26}^{1}$ and $D_{16}^{4}$, $D_{26}^{4}$ are zero.

(22) $$D=\left[\begin{array}{cccccc} 4.791\times 10^{5}N/\mathrm{mm} & 1.583\times 10^{5}N/\mathrm{mm} & 0 & 0 & 0 & 0\\ 1.583\times 10^{5}N/\mathrm{mm} & 4.791\times 10^{5}N/\mathrm{mm} & 0 & 0 & 0 & 0\\ 0 & 0 & 1.774\times 10^{5}N/\mathrm{mm} & 0 & 0 & 0\\ 0 & 0 & 0 & 12.778\times 10^{7}N\cdot \mathrm{mm} & 4.219\times 10^{7}N\cdot \mathrm{mm} & 0\\ 0 & 0 & 0 & 4.219\times 10^{7}N\cdot \mathrm{mm} & 12.778\times 10^{7}N\cdot \mathrm{mm} & 0\\ 0 & 0 & 0 & 0 & 0 & 4.497\times 10^{7}N\cdot \mathrm{mm} \end{array}\right]$$

Following the computing process shown in Figure 5, the equivalent shear stiffness of the considered lattice sandwich plate structure is

(23) $$K=\left[\begin{array}{cc} 1.603\times 10^{4}N/\mathrm{mm} & 0\\ 0 & 1.603\times 10^{4}N/\mathrm{mm} \end{array}\right]$$

The remainder part of this section discusses the effects of the unit cell parameters on the equivalent structural stiffness of the Bi-layer lattice cell.

#### 3.1.1. Effects of Unit Cell Parameters on Tensile Stiffness

**Figure 7:**
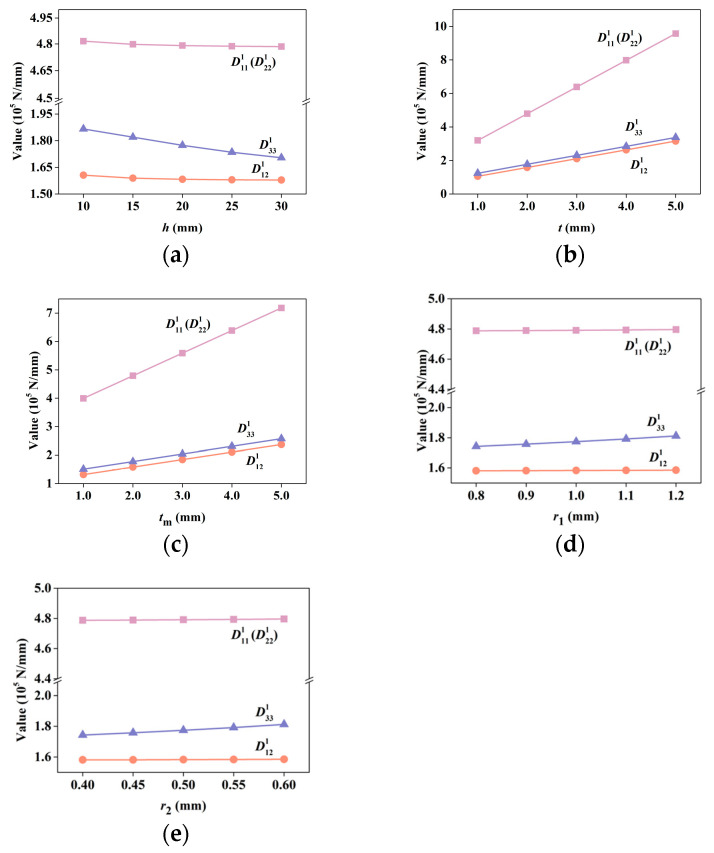
Tensile stiffness coefficients in ***D***^1^ by S-NIAH versus various unit cell parameters (*h*, *t*, *t*_m_, *r*_1_, and *r*_2_): (**a**) lattice height *h*, (**b**) upper and lower bound plate thickness *t*, (**c**) inner plate thickness *t*_m_, (**d**) radii of robs in spare layer *r*_1_, and (**e**) radii of robs in dense layer *r*_2_.

The variations in tensile stiffness ***D***^1^ versus various unit cell parameters are shown in Figure 7. Lattice height *h*, upper and lower bound plate thickness *t*, inner plate thickness *t*_m_, radii of the robs in the spare layer *r*_1_, and radii of the robs in the dense layer *r*_2_ are considered. It can be seen that *h*, *r*_1_, and *r*_2_ have a slight effect on the values of the coefficients in ***D***^1^, whereas *t* and *t*_m_ show large effect on them. This is because ***D***^1^ represents the tensile stiffness, and changing *t* and *t*_m_ directly changes the bearing area of the tensile force; thus, the variation in the thickness of the plates directly affects the values of the coefficients in ***D***^1^. In contrast, it can also be observed that the changes in the values of the coefficients in ***D***^1^ are linear, which is consistent with the correlation of the bearing area and the tensile stiffness.

#### 3.1.2. Effects of Unit Cell Parameters on Bending Stiffness

**Figure 8:**
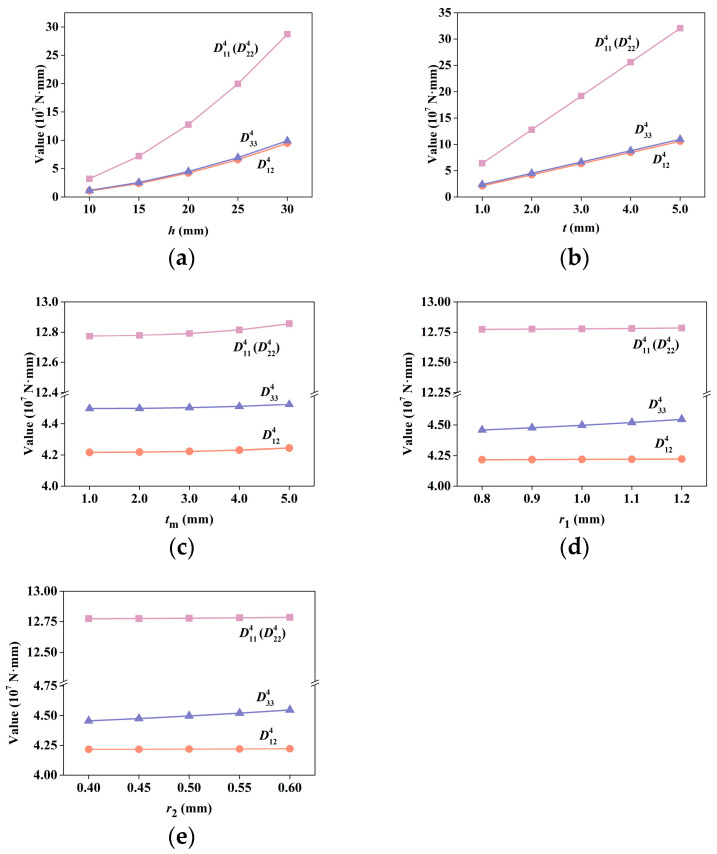
Bending stiffness coefficients in ***D***^4^ by S-NIAH versus various unit cell parameters (*h*, *t*, *t*_m_, *r*_1_, and *r*_2_): (**a**) lattice height *h*, (**b**) upper and lower bound plate thickness *t*, (**c**) inner plate thickness *t*_m_, (**d**) radii of robs in spare layer *r*_1_, and (**e**) radii of robs in dense layer *r*_2_.

The variations in tensile stiffness ***D***^4^ versus various unit cell parameters are shown in Figure 8. The considered parameters are the same as mentioned earlier. It can be seen that *h* and *t* have large effects on the values of the coefficients in ***D***^4^, whereas other parameters show little effect. ***D***^4^ represents the bending stiffness, and changing *h* and *t* directly changes the bearing area or the moment of inertia; thus, the variation in the lattice height thickness of the upper and lower bound plate directly affects the values of the coefficients in ***D***^4^. The difference is that the influence of *t* is linear, whereas that of *h* is nonlinear.

#### 3.1.3. Effects of Unit Cell Parameters on Shear Stiffness

**Figure 9:**
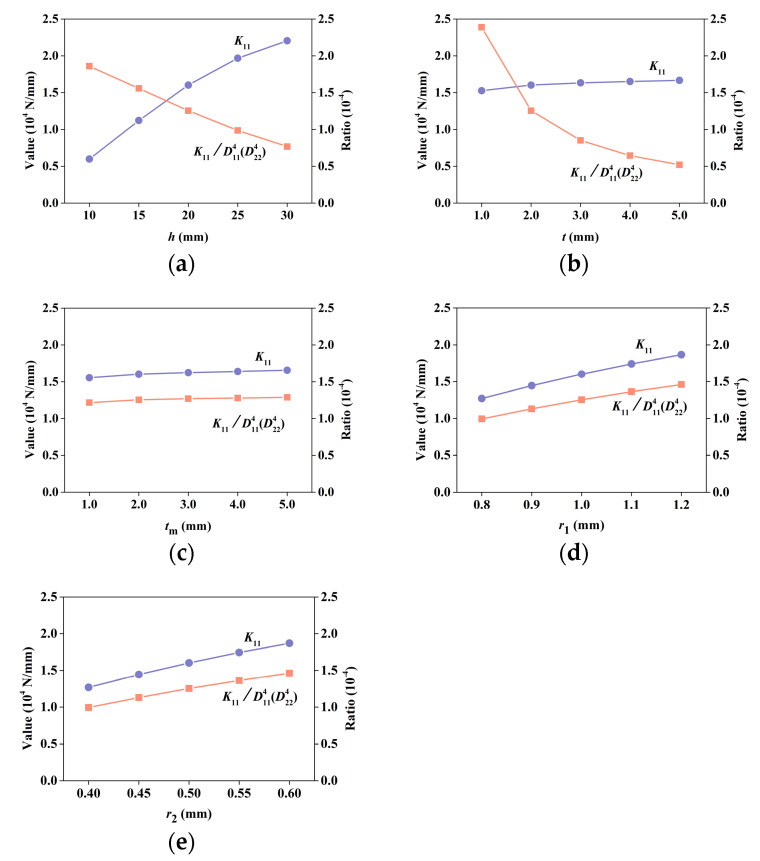
Shear stiffness coefficients in ***K*** by S-NIAH and ratio *K*_11_/$D_{11}^{4}$($D_{22}^{4}$) versus various unit cell parameters (*h*, *t*, *t*_m_, *r*_1_, and *r*_2_): (**a**) lattice height *h*, (**b**) upper and lower bound plate thickness *t*, (**c**) inner plate thickness *t*_m_, (**d**) radii of robs in spare layer *r*_1_, and (**e**) radii of robs in dense layer *r*_2_.

The variations in shear stiffness ***K*** versus various unit cell parameters are shown in Figure 9. The considered parameters are the same as mentioned earlier. The ratio of the shear stiffness to the bending stiffness is also shown in Figure 9. It can be seen that as the height increases, the shear stiffness increases, whereas ratio *K*_11_/$D_{11}^{4}$($D_{22}^{4}$) is reduced, which suggests that the bending stiffness increases more rapidly than the shear stiffness. As *t* increases, the shear stiffness shows little increase, whereas the ratio *K*_11_/$D_{11}^{4}$($D_{22}^{4}$) rapidly decreases. With the other parameters, the shear stiffness and ratio *K*_11_/$D_{11}^{4}$($D_{22}^{4}$) show the same trends of change. The results show that the contribution of the shear deformation to the total structural deformation rises when *h* or *t* increases, indicating an increase in the necessity of considering the shear deformation.

### 3.2. Bi-Layer Lattice-Filled Sandwich Plate

#### 3.2.1. Deformation Analysis of Bi-Layer Lattice-Filled Sandwich Plate

**Figure 10:**
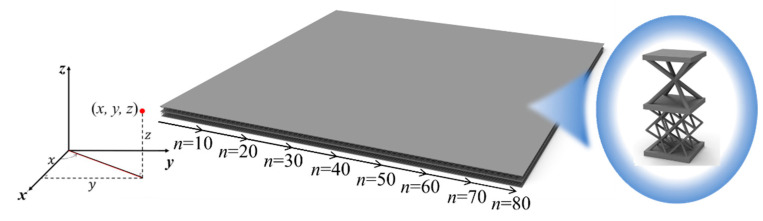
Model of Bi-layer lattice-filled sandwich plate.

**Figure 11:**
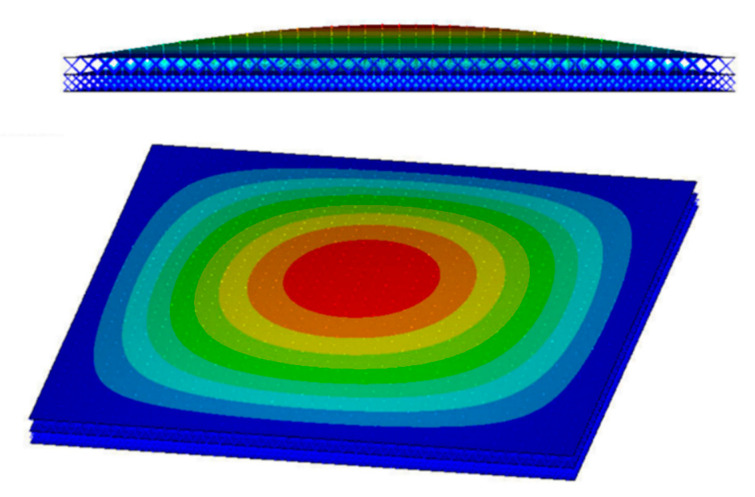
Displacement distribution of Bi-layer lattice-filled sandwich plate when *n* = 40.

The considered Bi-layer lattice-filled sandwich plate consists of *n* × *n* lattices, where *n* is the number of lattice extensions along *x* and *y* direction. The plate structure and the adopted lattice are shown in Figure 10. The four sides of the plate are fixed, and a unit uniform pressure is applied to the upper surface of the plate. Figure 11 shows the displacement distribution of the Bi-layer lattice-filled sandwich plate obtained by finite element modelling analysis when *n* = 40. 

**Figure 12:**
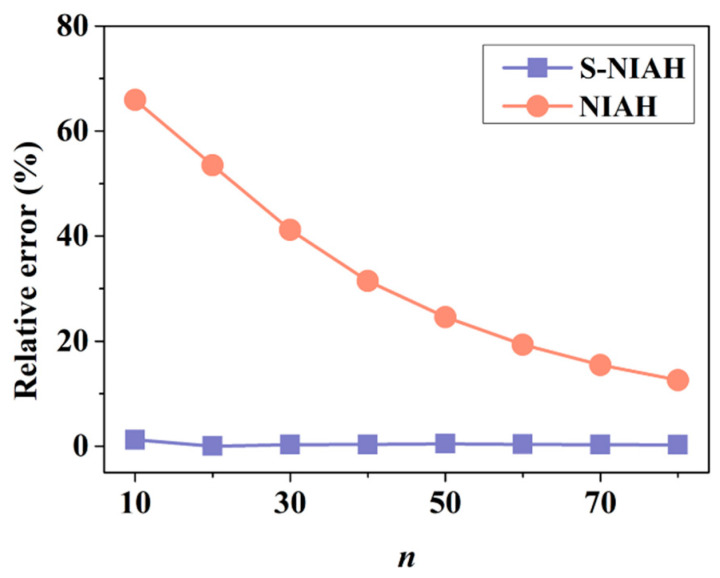
Relative error with respect to *n* under different methods.

To show the shear effect on the structural responses of the lattice structure, the structural responses are also obtained using the NIAH method. Table 1 lists the deformation results at the centre of the plate from both S-NIAH (the NIAH method considering equivalent shear stiffness) and NIAH (the NIAH method without considering equivalent shear stiffness) for different *n*. In the table, *w*_FEM_ represents the results from the finite element modelling analysis and *w*_S-NIAH_ and *w*_NIAH_ represent those using S-NIAH and NIAH, respectively. Figure 12 shows the variations in the errors of *w*_S-NIAH_ and *w*_NIAH_ with respect to *w*_FEM_ versus *n*.

It can be found that the results determined from S-NIAH are identical to those from the finite element modelling analysis even when *n* is small. The results obtained using NIAH show a large error when *n* is small, which keeps decreasing as *n* increases. The value of *n* is the number of lattice extensions in the structure, which affects the size of the plate. Because the parameters of the lattice are assumed to be constant, the length of the plate increases when *n* increases, leading to an increase in the length to thickness ratio. Therefore, the contributions of the shear effect to the global structural responses decreases. However, it can be seen that when *n* = 80, the error of *w*_NIAH_ is still much larger than that of *w*_S-NIAH_; thus, the shear effect should be concerned in Bi-layer lattice-filled plates.

The analysis results, considering the equivalent shear stiffness, are far superior to the equivalent analysis results without considering the equivalent shear stiffness under the same *n*. The errors of *w*_S-NIAH_ are in the range of engineering errors, whereas the errors of *w*_NIAH_ are far large to meet the engineering requirements. Therefore, for the Bi-layer lattice-filled sandwich plate structure, under the condition of limited number of extensions, the equivalent analysis considering the equivalent shear stiffness can yield higher prediction accuracy than the other method.

#### 3.2.2. Effects of Lattice Parameters on Accuracy of Results

This section studies the effects of other parameters on the errors of the results obtained from different NIAH methods. The value of *n* is set as 40.

The first considered parameter is the lattice height, *h*. The relative errors of the different methods are listed in Table 2. The results show that *w*_S-NIAH_ has high accuracy, whereas *w*_NIAH_ has low accuracy. It also can be seen that the value of *h* has little effect on the accuracy of the results.

Table 3 lists the relative errors of both methods versus the thickness, *t*. The results obtained from S-NIAH still demonstrate good performance, whereas those from NIAH still show poor performance. The results show that the thickness of the bound plate of the sandwich structure has a significant influence on the errors of the NIAH method. As *t* increases, the errors of the results from NIAH continuously increase. Specifically, as the thickness of the upper and lower plates increases, the contribution of the shear deformation to the global deformation increases.

Table 4, Table 5 and Table 6 list the errors of the results from both methods with different *t*_m_, *r*_1_, and *r*_2_, respectively. The trends are almost same as described above.

The above results again highlight the importance of the shear effect for the high-precision structural analysis of Bi-layer lattice-filled plates. However, as shown in Table 7, when the shear stiffness between the sparse and dense layer has a large difference, the responses from S-NIAH method will have relative larger errors.

In summary, the shear stiffness is mainly provided by the lattice core layer. By changing the parameters of the lattice core layer, the difference in shear stiffness between the sparse and dense layers is remarkable when the mass ratio of the sparse and dense layers increases. In view of the phenomenon that the disparity of the mass ratio increase causes the prediction error of the displacement response to increase, the mass ratio should be controlled to ensure the accuracy of the equivalent prediction in the structural analysis.

## 4. A Bi-Layer Lattice Filled Cylinder

### 4.1. The Equivalent Stiffness of Four Bi-Layer Lattice Cells

**Figure 13:**
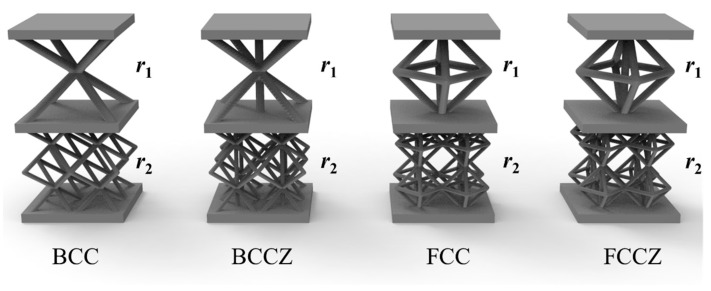
Structures of BCC, BCCZ, FCC and FCCZ lattice cells.

To further investigate the effectiveness of S-NIAH for Bi-layer lattice-filled structure, this section considers four different Bi-layer lattice cells, as shown in Figure 13.

Table 8 summarises the equivalent stiffness of the BCC, BCCZ, FCC, and FCCZ cells. For BCCZ, the radius of the sparse layer *r*_1_ is 0.9438 mm and the radius of the dense layer *r*_2_ is 0.4674 mm. For FCC, the radius of the sparse layer *r*_1_ is 0.9036 mm and the radius of the dense layer *r*_2_ is 0.4518 mm. For FCCZ, the radius of the sparse layer *r*_1_ is 0.8546 mm and the radius of the dense layer *r*_2_ is 0.4273 mm.

The results show that the stiffnesses differ remarkably in terms of the different types of lattice cells. Thus, the unit cell configuration has an important effect on the structural performance. FCC has greater tensile and bending stiffness than BCC, although the shear resistance is weaker compared to BCC. By adding supports in the Z-direction, the tensile and bending stiffnesses are slightly enhanced, whereas its shear stiffness is reduced and the shear resistance is weakened.

### 4.2. Bi-Layer Lattice-Filled Cylinder

The cylinder structure is widely used in engineering. Bi-layer lattices can help the cylinder structure to achieve high specific stiffness and high thermal insulation. However, owing to a lack of high-precision analysis methods, the structural design of gradient lattice-filled cylinders is difficult.

**Figure 14:**
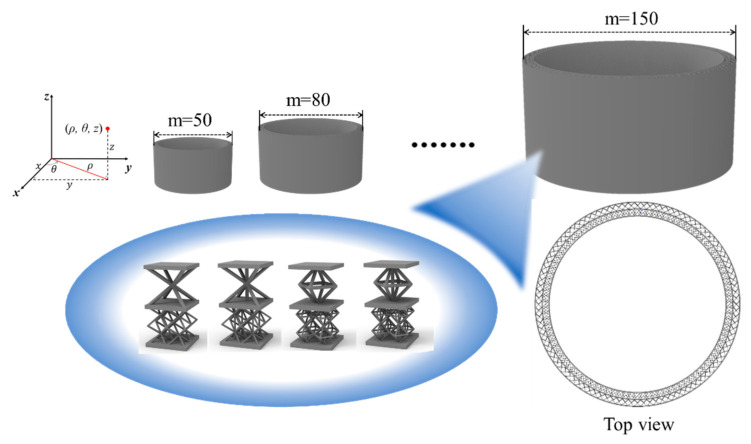
Model of Bi-layer lattice sandwich cylinder structure by different lattice cells.

A cylinder with an aspect ratio of 1:2 and a unit cell number *m* on the circumference shown in Figure 14 is used as an example to verify the validity of the equivalent analysis method for different lattice cells. The ends of the structure are fixed, and a unit uniform pressure is applied inside the cylinder for static analysis.

The node deflections along the thickness direction at an intermediate point with different lattice cells, *w*_FEM_, are compared to the mid-point deflection values considering the equivalent shear stiffness, *w*_S-NIAH_, and without considering the equivalent shear stiffness, *w*_NIAH_, under different *m*. For the BCC-filled Bi-layer lattice sandwich cylinder, the deflections at the mid-point of cylinder versus *m* from finite element modelling analysis, S-NIAH and NIAH, are shown in Table 9.

**Figure 15:**
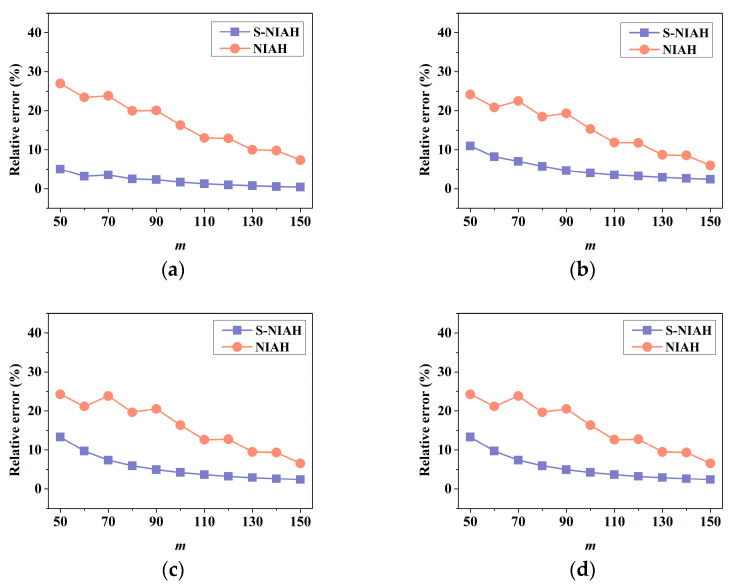
Relative error with respect to *m* under different methods (**a**) BCC; (**b**) BCCZ; (**c**) FCC; (**d**) FCCZ.

As shown in Figure 15, the analysis results considering the equivalent shear stiffness show high accuracy of the displacement response, whereas the results without the consideration of the shear effect present poor performance based on the precision analysis.

It can be seen that the difference in the lattice structure leads to the difference in structural responses by Table 9. The results show that when m is greater than 70, compared with the finite element modelling analysis, the relative error of the S-NIAH’s results is less than 10%, and as the number of extensions increases, the relative error is significantly reduced.

## 5. Conclusions

For gradient lattice-filled sandwich structures, there is a lack of high-precision numerical analysis methods. This study investigates using the newly proposed asymptotic homogenization method, NIAH, to overcome this problem. The Bi-layer lattice-filled sandwich structures are considered in numerical examples. The necessity of considering the shear effect is studied by comparing the accuracies of the structural responses obtained using the S-NIAH method developed based on the Mindlin plate theory and the NIAH method developed based on the Kirchhoff theory. The effects of the size effect, macrostructure type, and lattice type are also considered in the numerical examples. The numerical results show that the analysis results obtained from the S-NIAH method have good performance, based on accuracy analysis. From the examination of the structural parameters of the lattice proposed in this paper, it can be seen that when the mass ratio of the sparse and dense layers increases, the error in the prediction of the displacement responses increases. The verification of other lattice configurations confirms the applicability of the NIAH method considering the shear stiffness to the equivalent analysis of the displacement response of a Bi-layer gradient lattice structure.

In addition, based on this study, we find that for the considered Bi-layer lattice sandwich structure, an increase in the number of cells can reduce the error between the equivalent analysis results and the finite element modelling analysis results. In addition to increasing the number of extensions, another approach to reduce the error is to improve the unit cell shear resistance. When the ratio of the shear stiffness to bending stiffness is large, the unit cell has a strong shear resistance and the shear deformation does not have an extremely significant effect on the deformation mode of the structure. Conversely, the shear stiffness has a greater effect on the analysis results. Therefore, in engineering applications, the design parameters need to be adjusted when performing equivalently analysis because more lattice cells are filled on the macroscopic level. Moreover, the shear resistance of the unit cells should be improved on the microscopic level to ensure the accuracy of the equivalent analysis results. In addition, we will manufacture gradient lattice structures based on 3D printing technology and design experiments to further verify the accuracy of this method. We can also try to develop this method on the nano or micro scale and explore the performance of this method in the trans-scale field.

## Figures and Tables

**Table 1:** Average values of node deflection along thickness direction at centre *w*_FEM_, deflection values considering equivalent shear stiffness *w*_S-NIAH_, and deflection values without considering equivalent shear stiffness *w*_NIAH_ under different *n*.

n	w_FEM_ (mm)	w_S-NIAH_ (mm)	Error	w_NIAH_ (mm)	Error
10	0.019	0.020	1.23%	0.007	65.91%
20	0.100	0.101	0.01%	0.047	53.48%
30	0.301	0.302	0.30%	0.178	41.17%
40	0.719	0.721	0.34%	0.494	31.46%
50	1.462	1.468	0.44%	1.107	24.57%
60	2.734	2.744	0.37%	2.214	19.31%
70	4.728	4.742	0.30%	4.009	15.46%
80	7.689	7.708	0.25%	6.738	12.58%

**Table 2:** Deflections and errors at centre of plate versus *h*.

*h* (mm)	*w*_FEM_ (mm)	*w*_S-NIAH_ (mm)	Error
10	2.449	2.456	0.29%
15	1.169	1.170	0.09%
20	0.719	0.721	0.28%
25	0.513	0.516	0.58%
30	0.405	0.408	0.74%

**Table 3:** Deflections and errors at centre of plate versus *t*.

*t* (mm)	*w*_FEM_ (mm)	*w*_S-NIAH_ (mm)	Error
1.0	1.140	1.152	1.04%
2.0	0.719	0.721	0.28%
3.0	0.571	0.575	0.70%
4.0	0.493	0.501	1.62%
5.0	0.443	0.456	2.93%

**Table 4:** Deflections and errors at mid-point under different *t*_m_.

*t*_m_ (mm)	*w*_FEM_ (mm)	*w*_S-NIAH_ (mm)	Error
1.0	0.728	0.729	0.14%
2.0	0.719	0.721	0.28%
3.0	0.715	0.717	0.28%
4.0	0.711	0.712	0.14%
5.0	0.706	0.707	0.14%

**Table 5:** Deflections and errors at mid-point under different *r*_1_.

*r*_1_ (mm)	*w*_FEM_ (mm)	*w*_S-NIAH_ (mm)	Error
0.8	0.794	0.798	0.50%
0.9	0.752	0.753	0.13%
1.0	0.719	0.721	0.28%
1.1	0.694	0.696	0.29%
1.2	0.675	0.677	0.30%

**Table 6:** Deflections and errors at mid-point under different *r*_2_.

*r*_2_ (mm)	*w*_FEM_ (mm)	*w*_S-NIAH_ (mm)	Error
0.40	0.796	0.799	0.37%
0.45	0.752	0.754	0.27%
0.50	0.719	0.721	0.28%
0.55	0.694	0.696	0.29%
0.60	0.675	0.675	0.09%

**Table 7:** Deflections and errors at mid-point under *r*_1_ and *r*_2_.

Mass Ratio	Dimensions (mm)		Shear Stiffness (10^2^ N/mm)		*w*_FEM_ (mm)	*w*_S-NIAH_ (mm)	Error
	*r*_1_	*r*_2_	*r*_1_	*r*_2_			
90:10	1.342	0.224	30.280	0.891	1.015	1.215	19.70%
70:30	1.183	0.387	18.550	7.128	0.762	0.773	1.44%
50:50	1.000	0.500	9.587	17.872	0.719	0.721	0.28%
30:70	0.775	0.592	3.796	31.952	0.758	0.772	1.85%
10:90	0.447	0.671	0.394	48.596	0.999	1.213	21.42%

**Table 8:** Equivalent stiffness coefficients of different lattice cells.

Configuration	$D_{11}^{1}$ **(10^5^ N/mm)**	$D_{12}^{1}$ **(10^5^ N/mm)**	$D_{22}^{1}$ **(10^5^ N/mm)**	$D_{33}^{1}$ **(10^5^ N/mm)**	$D_{11}^{4}$ **(10^7^ N•mm)**
BCC	4.791	1.583	4.791	1.774	12.778
BCCZ	4.847	1.640	4.847	1.752	12.847
FCC	4.979	1.643	4.979	1.731	12.990
FCCZ	4.981	1.659	4.981	1.717	12.991
**Configuration**	$D_{12}^{4}$ **(** **10^7^** **N•mm)**	$D_{22}^{4}$ **(** **10^7^** **N•mm)**	$D_{33}^{4}$ **(** **10^7^** **N•mm** **)**	$K_{11}$ **(** **10^4^** **N/mm)**	$K_{22}$ **(** **10^4^** **N/mm)**
BCC	4.219	12.778	4.497	1.603	1.603
BCCZ	4.290	12.847	4.469	1.441	1.441
FCC	4.285	12.990	4.422	1.208	1.208
FCCZ	4.303	12.991	4.407	1.093	1.093

**Table 9:** Average values of node deflection at mid-point of cylinder *w*_FEM_, deflection values considering equivalent shear stiffness *w*_S-NIAH_, and deflection values without considering equivalent shear stiffness *w*_NIAH_ under different *m*.

Lattice Cell	*m*	*w*_FEM_ (10^−2^ mm)	*w*_S-NIAH_ (10^−2^ mm)	Error	*w*_NIAH_ (10^−2^ mm)	Error
BCC	50	0.455	0.478	5.02%	0.332	27.00%
	60	0.682	0.704	3.21%	0.522	23.44%
	70	0.911	0.943	3.54%	0.694	23.85%
	80	1.229	1.259	2.51%	0.983	19.97%
	90	1.543	1.579	2.32%	1.234	20.05%
	100	1.957	1.991	1.70%	1.637	16.33%
	110	2.421	2.452	1.27%	2.106	13.03%
	120	2.878	2.907	0.99%	2.506	12.91%
	130	3.443	3.471	0.78%	3.099	9.99%
	140	3.995	4.017	0.56%	3.604	9.80%
	150	4.664	4.684	0.44%	4.321	7.35%
BCCZ	50	0.435	0.482	10.93%	0.330	24.19%
	60	0.655	0.708	8.21%	0.518	20.85%
	70	0.888	0.950	7.04%	0.688	22.50%
	80	1.196	1.265	5.75%	0.975	18.49%
	90	1.516	1.587	4.67%	1.223	19.33%
	100	1.917	1.994	4.04%	1.623	15.35%
	110	2.367	2.451	3.56%	2.086	11.84%
	120	2.815	2.906	3.25%	2.484	11.76%
	130	3.363	3.461	2.92%	3.070	8.71%
	140	3.905	4.008	2.64%	3.571	8.57%
	150	4.553	4.664	2.43%	4.280	6.00%
FCC	50	0.426	0.483	13.32%	0.322	24.27%
	60	0.643	0.705	9.71%	0.507	21.19%
	70	0.884	0.949	7.38%	0.673	23.84%
	80	1.186	1.257	5.96%	0.953	19.66%
	90	1.505	1.581	4.96%	1.196	20.52%
	100	1.897	1.977	4.25%	1.586	16.35%
	110	2.334	2.420	3.71%	2.039	12.65%
	120	2.782	2.872	3.22%	2.427	12.76%
	130	3.312	3.408	2.90%	2.998	9.48%
	140	3.846	3.947	2.63%	3.487	9.34%
	150	4.471	4.579	2.42%	4.178	6.56%
FCCZ	50	0.426	0.488	24.27%	0.323	24.31%
	60	0.642	0.712	21.19%	0.507	21.03%
	70	0.883	0.959	23.84%	0.673	23.76%
	80	1.184	1.268	19.66%	0.954	19.47%
	90	1.504	1.595	20.52%	1.197	20.42%
	100	1.893	1.993	16.35%	1.587	16.15%
	110	2.327	2.436	12.65%	2.039	12.36%
	120	2.776	2.891	12.76%	2.428	12.54%
	130	3.302	3.426	9.48%	2.999	9.17%
	140	3.836	3.968	9.34%	3.488	9.07%
	150	4.456	4.597	6.56%	4.180	6.20%

## Data Availability

Not applicable.

## References

[1] Wadley H.N.G., Fleck N.A., Evans A.G. (2003). Fabrication and structural performance of periodic cellular metal sandwich structures. Compos. Sci. Technol..

[2] Ashby M.F., Evans A., Fleck N.A., Gibson L.J., Hutchinson W.J., Wadley H.N.G. (2012). Metal foams: A design guide. Appl. Mech. Rev..

[3] Gibson L.J. (2003). Cellular solids. Mrs. Bull..

[4] Zheng J., Qin Q., Wang T.J. (2016). Impact plastic crushing and design of density-graded cellular materials. Mech. Mater..

[5] Nasirov A., Fidan I. (2020). Prediction of mechanical properties of fused filament fabricated structures via asymptotic homogenization. Mech. Mater..

[6] Silva E., Walters M.C., Paulino G.H. (2006). Modeling bamboo as a functionally graded material: Lessons for the analysis of affordable materials. J. Mater. Sci..

[7] Meng L., Zhang W., Quan D., Shi G.H., Gao T. (2021). Correction to: From Topology Optimization Design to Additive Manufacturing: Today’s Success and Tomorrow’s Roadmap. Arch. Comput. Methods E.

[8] Hu J., Luo Y., Liu S. (2021). Two-scale concurrent topology optimization method of hierarchical structures with self-connected multiple lattice-material domains. Compos. Struct..

[9] Li Q.H., Xu R., Wu Q.B., Liu S.T. (2021). Topology optimization design of quasi-periodic cellular structures based on erode–dilate operators. Comput. Method. Appl. Mech. Eng..

[10] Yan J., Zhang C.G., Huo S.X., Chai X.H., Liu Z.H., Yan K. (2021). Experimental and numerical simulation of bird-strike performance of lattice-material-infilled curved plate. Chin. J. Aeronaut..

[11] Sun Y., Guo L.C., Wang T.S., Zhong S.Y., Pan H.Z. (2017). Bending behavior of composite sandwich structures with graded corrugated truss cores. Compos. Struct..

[12] Zhang J., Wang Z., Zhao L. (2016). Dynamic response of functionally graded cellular materials based on the Voronoi model. Compos. Part B.

[13] Lefebvre J.E., Zhang V., Gazalet J., Gryba T., Sadaune V. (2001). Acoustic wave propagation in continuous functionally graded plates: An extension of the Legendre polynomial approach. IEEE T Ultrason. Ferroelectr. Freq. Control.

[14] Sun G., Wang E., Wang H., Xiao Z., Li Q. (2018). Low-velocity impact behaviour of sandwich panels with homogeneous and stepwise graded foam cores. Mater. Design..

[15] Zhou J., Guan Z.W., Cantwell W.J. (2013). The impact response of graded foam sandwich structures. Compos. Struct..

[16] Woodward B., Kashtalyan M. (2011). 3D elasticity analysis of sandwich plates with graded core under distributed and concentrated loadings. Int. J. Mech. Sci..

[17] Liu X.R., Tian X.G., Lu T.J., Liang B. (2014). Sandwich plates with functionally graded metallic foam cores subjected to air blast loading. Int. J. Mech. Sci..

[18] Xu G.D., Zhai J.J., Tao Z., Wang Z.H., Fang D.N. (2015). Response of composite sandwich beams with graded lattice core. Compos. Struct..

[19] Guo L.C., Noda N. (2007). Modeling method for a crack problem of functionally graded materials with arbitrary properties—Piecewise-exponential model. Int. J. Solids Struct..

[20] Ajdari A., Nayeb-Hashemi H., Vaziri A. (2011). Dynamic crushing and energy absorption of regular, irregular and functionally graded cellular structures. Int. J. Solids Struct..

[21] Cheng L., Bai J., To A.C. (2019). Functionally graded lattice structure topology optimization for the design of additive manufactured components with stress constraints—ScienceDirect. Comput. Method. Appl. Mech. Eng..

[22] Li H., Luo Z., Zhang N., Gao L., Brown T. (2016). Integrated design of cellular composites using a level-set topology optimization method. Comput. Method. Appl. Mech. Eng..

[23] Cheng G.D., Cai Y.W., Xu L. (2013). Novel implementation of homogenization method to predict effective properties of periodic materials. Acta Mech. Sinica.

[24] Wang B., Tian K., Hao P., Zheng Y., Ma Y., Wang J. (2016). Numerical-based smeared stiffener method for global buckling analysis of grid-stiffened composite cylindrical shells. Compos. Struct..

[25] Kalamkarov A.L., Kolpakov A.G. (1997). Analysis, Design, and Optimization of Composite Structures.

[26] Cai Y., Xu L., Cheng G.D. (2014). Novel numerical implementation of asymptotic homogenization method for periodic plate structures. Int. J. Solids Struct..

[27] Xu L., Cheng G.D. (2017). Shear stiffness prediction of reissner-mindlin plates with periodic microstructures. Mech. Adv. Mater. Struc..

[28] Yan J., Jiang C.C., Fan Z.R., Xu Q., Du H.Z., Sun W., Wang G.Y., Niu B. (2021). Compression Experiment and Failure Analysis of Additive Manufactured Multi-Layer Lattice Sandwich Structure. Int. J. Appl. Mech..

